# Topical hyaluronic acid and oxygen for urinary incontinence: a double-center retrospective study

**DOI:** 10.3389/fmed.2026.1739601

**Published:** 2026-02-02

**Authors:** Paolo Manna, Basilio Pecorino, Fabiola Perrone, Marika Di Blasi, Paolo Scollo, Giuseppe Scibilia, Fulvio Zullo

**Affiliations:** 1 Department of Clinical and Experimental Medicine, Obstetrics and Gynecology Unit, “Magna Graecia” University, Catanzaro, Italy; 2 Obstetrics and Gynecology Umberto I Hospital, Kore University of Enna, Enna, Italy; 3 Obstetrics and Gynecology Cannizzaro Hospital, Kore University of Enna, Catania, Italy; 4 Obstetrics and Gynecology Giovanni Paolo II Hospital, Kore University of Enna, Ragusa, Italy

**Keywords:** genitourinary syndrome, hyaluronic acid and high concentration oxygen, menopause, mixed urinary incontinence, stress incontinence

## Abstract

**Purpose:**

This study aimed to evaluate the effects of low-molecular-weight hyaluronic acid (LMWHA) solution and high-concentration oxygen (HCO) through a specific medical device (Caress Flow©), compared to topical administration of hyaluronic acid (HA) alone, on symptoms of mild and moderate stress and mixed urinary incontinence in women with genitourinary syndrome of menopause (GSM).

**Methods:**

A total of 68 postmenopausal women were registered (from October 2021 to September 2023). Patients were divided into two groups: one group received therapy with LMWHA solution and HCO combined, and the second group received HA therapy alone. The primary outcome was the Patient Global Impression of Improvement (PGI-I). Secondary outcomes included the International Consultation on Incontinence Questionnaire–short form (ICIQ-SF) questionnaire and the International Quality of Life (IQoL) questionnaire.

**Results:**

Of the 68 women, 35 (51%) and 33 (49%) were treated with LMWHA solution and HCO combined and HA alone for 10 weeks, respectively. A total of 28 (80%) vs. 7 (21%) women reported an improvement (PGI-I score ≤3) of the symptoms in the LMWHA solution and HCO combined group vs. the HA alone group. According to the ICIQ-SF, a statistically significant difference was observed between the two groups after treatment (median, 7 [5–11] vs. 10 [8–11]; *p* = 0.03). The IQoL questionnaire recorded a statistically significantly lower median score in the LMWHA solution and HCO combined group compared with the HA group, before (71 [IQR 55–81] vs. 89 [IQR 67–94]; *p* < 0.01) and after (78 [IQR 65–86] vs. 88 [IQR 72–99]; *p* = 0.04) treatment.

**Conclusion:**

LMWHA solution and HCO combined might improve mild and moderate stress and mixed urinary incontinence in women with GSM and appear superior to HA therapy alone. However, these results need to be confirmed in further studies with a controlled design and a larger population.

## 1 Introduction

“Genitourinary Syndrome of Menopause” (GSM) is a chronic condition characterized by a group of signs and symptoms associated with absence of estrogens (ER) and other sex steroids caused by the cessation of ovarian function, involving the lower genital structures (labia majora/minora, clitoris, vestibule, vagina, and vulva) as well as the lower part of the urinary tract (specifically the bladder trigone and proximal urethra) (1). GSM is common, affecting approximately 50 to 70% of post-menopausal women, yet remains frequently underdiagnosed and undertreated. Symptoms related to GSM, especially vulvovaginal symptoms, interfere with sexual satisfaction (74%), spontaneity (70%), and partner relationships (66%) (2). These manifestations lead to discomfort during sexual intercourse, which is often avoided, further worsening atrophy and consequently causing a negative impact on the couple’s relationship and overall quality of life (3). In Italy, prevalence is comparable to that of other European countries. In “The AGATA Study” by F. Palma et al., 79.1% of post-menopausal women involved in the study were affected with GSM, with prevalence varying from 64.7% 1 year from menopause to 84.2% in the following 6 years (4). Hypoestrogenism following menopause represents the main pathogenetic cause of GSM, but there are several concomitant risk factors (5). Regarding the pathophysiology of urinary signs and symptoms of GSM, it was seen that the urethra becomes atrophic and prominent with a relative increase of transitional cells and a reduction of intermediate and superficial squamous cells. There is a remodeling of the bladder with a reduction of the tropism of smooth muscle tissue and collagen. This involves a reduction in the contractile force of the detrusor, bladder capacity and sensitivity, urethral closure pressure, and urinary flow (6, 7). The absence of ER receptors at the trigone reduces bladder sensory threshold and pressure to leakage, resulting in urgency incontinence and bladder hyperactivity (8). Collagen loss also increases laxity of pelvic floor support structures, contributing to prolapse of genitals and urinary incontinence. There are currently various therapeutic options to choose from, based on the patient’s clinical history and the severity of the symptoms: non-hormonal therapy, hormonal therapy, and laser therapy (9). Several studies have emphasized how topical oxygen therapy is useful in counteracting inflammatory dermatological diseases, androgenic alopecia, and dry eyes (10–12). In the literature, it is indicated that oxygen, in addition to having anti-inflammatory and antibacterial properties, would stimulate collagen synthesis and promote hydroxylation. Increasing oxygen may promote the release of vascular endothelial growth factor (VEGF), increasing the angiogenic stimulus and trophism of tissues (13, 14). Hyaluronic acid (HA) is a polymer normally found in the connective tissue known for its supporting, hygroscopic, re-epithelizing, viscoelastic, antioxidant, bacteriostatic, and anti-inflammatory properties. Administration of HA has proven effective in improving dryness, dyspareunia, and tropism of the vaginal epithelium in women affected by GSM and has also proven to be a valid alternative to conjugated estrogen (ER) therapy, also having effects on the symptoms of incontinence (15).

## 2 Materials and methods

### 2.1 Study design

This double-centered, retrospective study was conducted in the gynecological clinic of the Hospital “Pugliese-Ciaccio” in Catanzaro and “Umberto I” in Enna. The protocol was approved by the Ethical Committee of the Calabria Region (nr 342, October 2021). Menopausal patients with genitourinary syndrome of menopause with mild and moderate stress and mixed urinary incontinence were enrolled from October 2021 to September 2023. Patients were divided into two groups. The first group received combined therapy with low-molecular-weight hyaluronic acid (LMWHA) solution and high-concentration oxygen (HCO) through a specific device (Caress Flow©). The second group received topical daily therapy with vaginal tablets of HA for 10 weeks.

### 2.2 Inclusion criteria and validated questionnaire

Women with mild or moderate stress and mixed urinary incontinence, after verifying inclusion criteria, included good general health; age between 45 and 70 years; natural or surgical menopause at least 12 months before enrollment; absence of lower urinary tract infections (UTIs); absence of pre-existing prolapse; absence of incontinence due to neurological damage; absence of neoplasm affecting the genitourinary system; and absence of contraindications to local therapy with HCO and HA. The exclusion criteria included prolapse; incontinence due to neurological damage; genitourinary cancer; previous radio/brachytherapy; systemic intake of estrogens, progestins, androgens, selective modulators of estrogen receptor (SERM), intrauterine progestins, or vaginal lubricants and moisturizers within 8 weeks; alternatively taking transdermal or vaginal hormonal products within 4 weeks; signs and symptoms of severe urinary incontinence or absence of signs and/or symptoms of urinary incontinence; no previous gynecological surgeries nor surgery to treat urinary incontinence symptoms (such as TOT, TVT, or Burch procedure).

Patients were selected based on the scores obtained from the questionnaires administered during the first outpatient visit. The questionnaires were approved by the International Consultation on Incontinence (ICI) and validated in the Italian language. Using the overactive bladder screener (OAB-S), the presence of urge incontinence symptoms was evaluated, and with the International Consultation on Incontinence Questionnaire–Short Form (ICIQ-SF), the frequency and severity of urinary leakages were assessed, how much these leaks impacted daily activities, and when they appeared. It is composed of three questions plus a multiple-choice question about urine leaks. The total score allows classifying incontinence as mild (scores 1–5), moderate (scores 6–12), severe (scores 13–18), and very severe (scores 19–21). Only women with mild or moderate stress or mixed incontinence (ICIQ-SF score ≤12) were enrolled. The OAB-S is composed of eight questions, with scores ranging from 8 to 48, with a score >8 representating the presence of incontinence. This questionnaire was repeated at the end of five sessions to verify symptomatic improvements. Further, the International Quality of Life (IQoL) questionnaire is a validated tool composed of 22 questions that assess the impact of incontinence on quality of life. Total scores range from 22 to 110. The lower score indicates the stronger impact of urinary incontinence on quality of life. At the end of five treatment sessions, the Patient Global Impression of Improvement (PGI-I) questionnaire was administered. The PGI-I questionnaire is composed of a single question evaluating the perceived improvement by the patient of a specific symptom after treatment, with scores ranging from 1 (meaning “very much better”) to 7 (meaning “very much worse”). An improvement is defined when the PGI-I score is ≤3. At this point, the women were also asked to answer the ICIQ-SF and the IQoL questionnaires again. All participants provided written informed consent prior to enrollment.

### 2.3 Treatment administration

**Figure 1:**
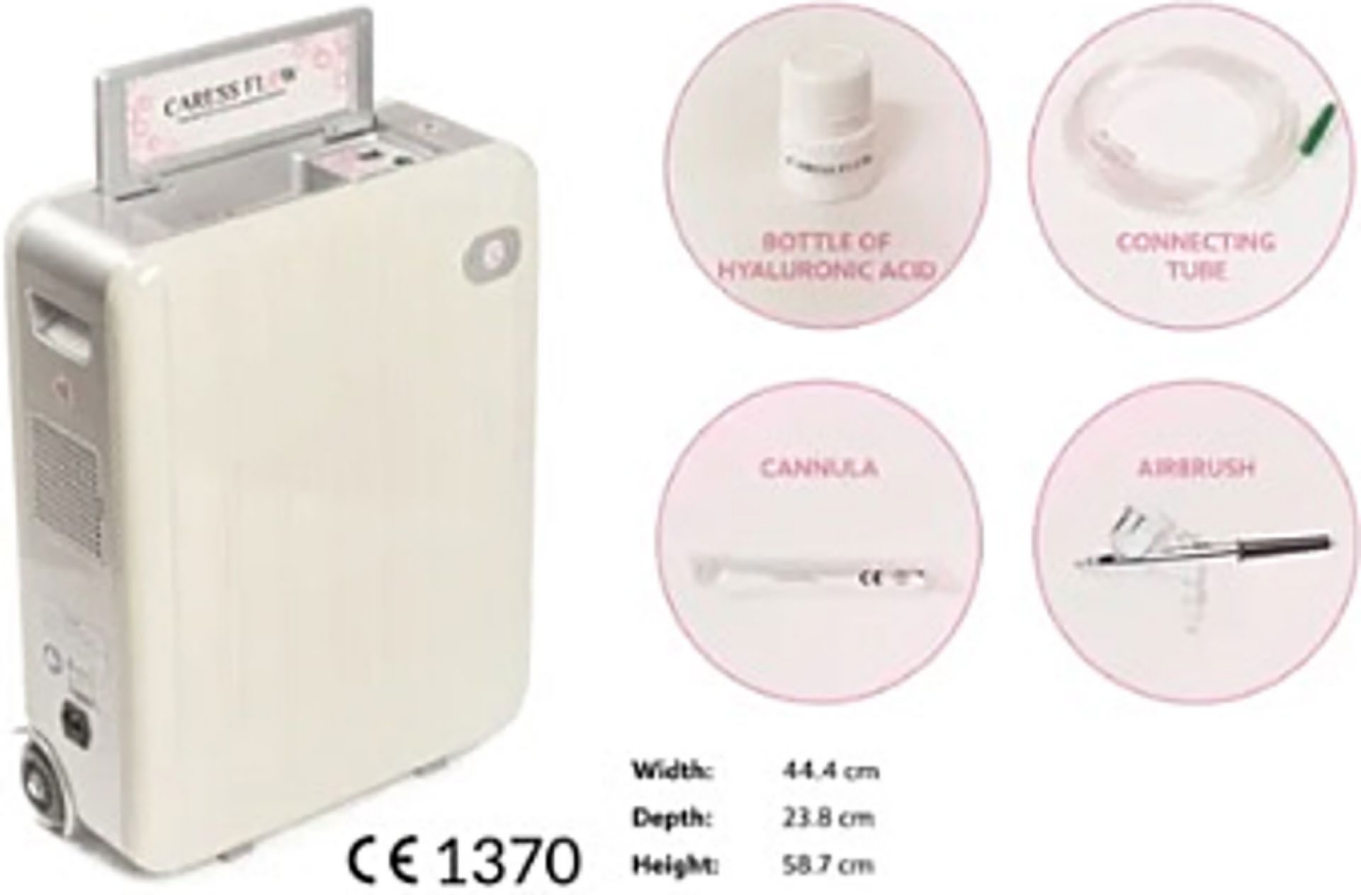
Disposable kit containing low-molecular-weight hyaluronic acid from Caress Flow with an electronic device delivering high-concentration oxygen.

Patients underwent, under medical prescription, combined therapy with LMWHA solution and HCO for 15 min a session, for a total of five sessions at 14-day intervals (for a total of 10 weeks), using the *X2 self-cooling device (Caress Flow©)* (Figure 1) or hyaluronic acid (HA) vaginal tablet therapy (dose is the same as 1 vaginal tablet for 10 weeks every night).

The procedure was performed in an outpatient setting with patients placed in a lithotomy position. After gentle cleansing of the vulvar area, the single-use applicator was inserted into the vaginal canal without the need for speculum use or cervical dilation. Once positioned, the applicator was activated to allow the controlled release of the oxygen flow together with the HA gel. The tip of the applicator was kept in place for 15 min, ensuring uniform distribution of the formulation along the vaginal walls. At the end of the administration, the applicator was removed and discarded. Patients were advised to avoid intravaginal products or sexual intercourse for the subsequent 24 h to prevent interference with the absorption of the active components. No analgesia or anesthesia was required, and the procedure was well tolerated by all participants.

### 2.4 Effectiveness evaluation procedures

For both groups, LMWHA solution and HCO combined and HA alone, 30 days after the treatment a follow-up visit was performed and on that occasion the following questionnaires were administered to evaluate the possible improvement of urinary symptoms: the primary outcome used PGI-I to study the degree of improvement perceived by the patient following the five-session treatment in the LMWHA solution and HCO combined group in the comparison with the local HA group. Secondary outcomes included OAB-S, ICIQ-SF, and IQoL. During each session, any adverse event was noted and reported according to the criteria of CTGAE.

### 2.5 Statistical analyses

The primary endpoint was the difference in the proportion of women who reported improvements (PGI-I scores ≤3) between the two groups. Secondary endpoints were ICIQ-SF and IQoL scores at the end of five treatment sessions. Descriptive statistics were presented as means and SDs, medians, and interquartile ranges (IQRs) for continuously coded variables or counts and percentages for categorically coded variables. Furthermore, the *t*-test, Kruskal–Wallis test, and χ2 test examined the statistical significance of differences between means, medians, and proportions. A statistical power analysis was performed to evaluate the adequacy of the sample size for detecting clinically meaningful differences between the groups. The Wilcoxon signed-rank test for the paired sample was performed to compare continuous non-parametric variables. In all statistical analyses, the R software (University of Toronto, Canada) environment for statistical computing and graphics (R version 4.0.0) was used. All tests were two-sided with a level of significance set at a *p*-value of < 0.05.

## 3 Results

From October 2021 to September 2023, a total of 68 postmenopausal women were enrolled. Among them, 35 (51.5%) had started and completed combined therapy with LMWHA solution and HCO combined, and 33 (48.5%) had used HA therapy. In the overall population (Table 1), the median age was 59.7 years (IQR, 45–66 years), and the median menopause age was 58.5 years (IQR, 45–64 years). There was no statistically significant difference in age (*p* = 0.4) and age at menopause (*p* = 0.4) between the two groups. All women enrolled reported an OAB-S score >8 (overall median score, 28 [IQR, 22.5–34]), and no statistically significant difference was recorded between the two groups, *p* = 0.9. All women were Caucasian of Italian ancestry.

**Table 1:** Selection criteria and study population characteristics.

Variables	HCO + LMWH combined	HA topically alone	*p*-value
Age	59.7	58.5	0.46
BMI	24.7	25.0	0.47
Parity	2.1	1.8	0.78
LMP (y)	50.2	51.2	0.12
Distance from LMP (y)	5.5	6.0	0.08
IQoL score pre-treatment	73	77	0.91
OAB score pre-treatment	27	26	0.34

### 3.1 Primary endpoint: PGI-I questionnaire at 10 weeks after treatment

**Figure 2:**
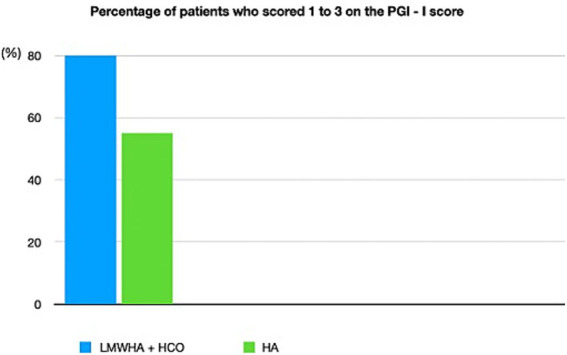
Patient global impression of improvement (PGI-I) questionnaire was administered to 35 women (51%) who received LMWHA solution and HCO combined and 33 women (49%) who received HA therapy after 10 weeks of treatment. The PGI-I questionnaire stratified in ≤3 and >3 scores. The PGI-I questionnaire reports all possible answer results.

According to the PGI-I questionnaire (Figure 2), a significantly higher percentage of women reported an improvement (score ≤3) in the LMWHA solution and HCO combined group than in the hyaluronic acid (HA) alone group (*p* < 0.001). Indeed, 28 women (80%) in the LMWHA solution and HCO combined group reported an improvement at the end of treatment (2 [7.1%] very much better, 7 [25%] much better, and 19 [67.9%] a little better). Conversely, 18 (55%) women in the HA group reported no improvement at the end of the treatment (15 [83.4%] no change, 2 [11.1%] a little worse, and 1 [5.5%] much worse).

### 3.2 Secondary endpoint I: ICIQ-SF questionnaire

**Figure 3:**
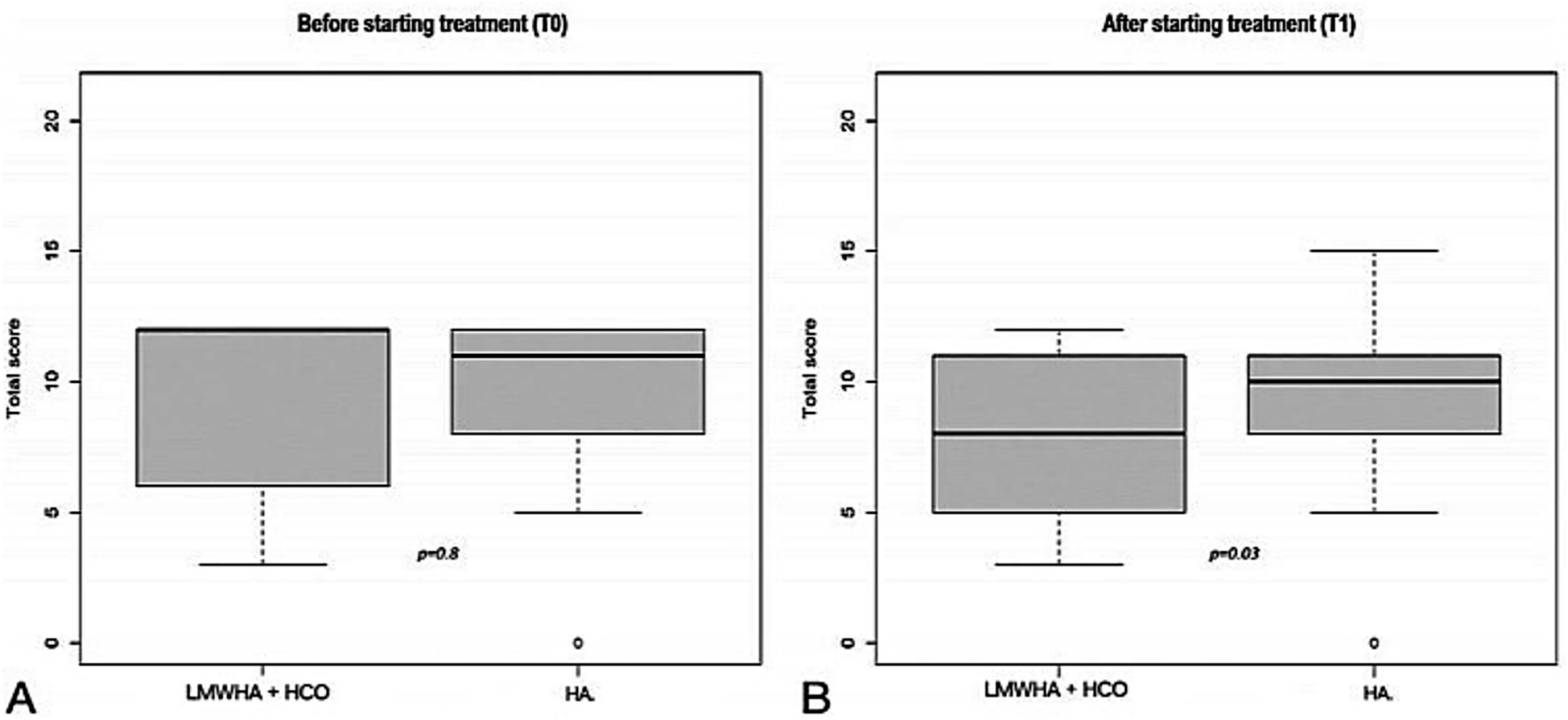
International Consultation on Incontinence (ICIQ-SF) questionnaire before (T0, **A**) and after (T1, **B**) starting treatment, between the LMWHA solution and HCO combined and HA group. Boxes denote the interquartile range. The solid black horizontal bar denotes the median score. Whiskers denote the 95% range of the distribution of the ICIQ-SF score. The open circles denote outlier values.

According to the ICIQ-SF questionnaire (Figure 3), in the between-group analysis, no statistically significant difference was recorded before treatment administration between the LMWHA solution and HCO combined and HA alone groups (median score, 12 [6–12] vs. 10 [8–12]; *p* = 0.8). Conversely, a statistically significant difference was recorded after treatment administration between the two groups (median score, 7 [5–11] vs. 10 [8–11]; *p* = 0.03).

### 3.3 Secondary endpoint II: IQoL questionnaire

**Figure 4:**
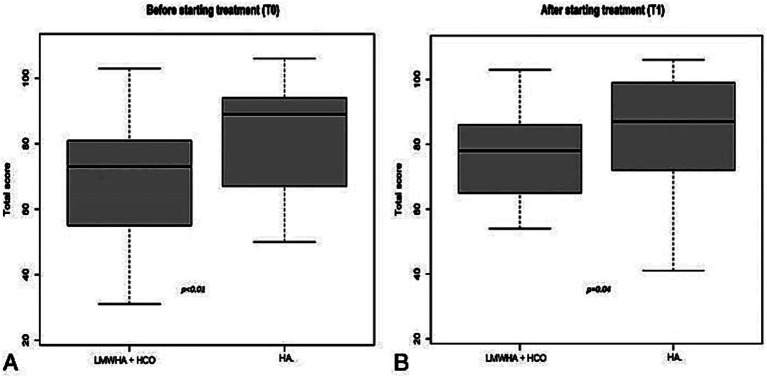
International Quality of Life (IQoL) questionnaire was administered before (T0, **A**) and after (T1, **B**) starting treatment, between the LMWHA solution and the HCO combined group and the HA group. Boxes denote the interquartile range. The solid black horizontal bar denotes the median score. Whiskers denote the 95% range of the distribution of the IQoL score.

According to the IQoL questionnaire (Figure 4), in the between-group analysis, a statistically significantly lower median score was documented in the LMWHA solution and HCO combined group than the HA alone group, before (71 [IQR 55–81] vs. 89 [IQR 67–94]; *p* < 0.01) and after (78 [IQR 65–86] vs. 88 [IQR 72–99]; *p* = 0.04) treatment.

## 4 Discussion

The prevalence of bladder symptoms, such as frequency, urgency, and incontinence, tends to increase around the menopause, although it is still debated whether these disorders are due to menopause, aging, or a combination of the two. Furthermore, multiparity and obesity are well-known risk factors for urinary incontinence. Regarding obesity, it is a risk factor associated with bladder incontinence (16). It is interesting to note that both study populations have a high BMI compared with the norm, but with a BMI belonging to the same range (24.9–29.99) without a statistically significant difference between the groups. Obesity seems to act according to a mechanical mechanism of increased intravisceral pressure with discomfort of the pelvic muscles, as an inflammatory factor produced by lipokines and proinflammatory agents generated by adipose tissue, and with a metabolic hormone mechanism with consequent alteration of the glycemic homeostasis impacting on the urogenital microbiome (16). Urinary symptoms are associated with GSM, which is characterized by physical and hormonal modifications. It has been seen that hormonal therapies improve the symptoms of GSM, but to date, their time of use/dose and the type of effect on long-term systemic levels are still unclear. Local ES therapy has played and plays an important role in maintaining good tropism of the vaginal mucosa, but even in this case, not all patients accept it, and, in some situations, the effectiveness might not be optimal. Furthermore, the impossibility of hormonal therapies’ use in some categories of women with high-risk factors, such as previous breast cancer or thromboembolic diseases, must be considered (17). The use of non-hormonal therapies that significantly improve the symptoms of GSM and the quality of life of affected patients is successful, but many topical, non-hormonal products on the market have demonstrated a temporary positive action in promoting only lubrication but are often not sufficient to solve all the problems related to mild and moderate stress or mixed urinary incontinence of GSM but allow only transient benefits about vaginal trophism, with the consequent need for repeated treatments (18, 19). Several studies support the use of non-hormonal therapies for the treatment of stress and mixed incontinence in postmenopausal women. In particular, several studies have highlighted how topical oxygen therapy is useful in counteracting inflammatory diseases because the oxygen reactivates the microcirculation of the urogenital epithelium, favoring neoangiogenesis, stimulating cellular turnover, improving trophism, and conveying nutrients to the epithelial cells. The combined application of LMWHA and topical oxygen exerts a synergistic effect on mucosal regeneration in women with genitourinary syndrome of GSM. LMWHA contributes to epithelial hydration, extracellular matrix remodeling, and fibroblast activation, ultimately supporting collagen deposition and restoration of mucosal elasticity (13). Oxygen therapy, on the other hand, has been shown to improve angiogenesis through the upregulation of VEGF, enhance cellular respiration, and stimulate fibroblast proliferation, thereby increasing collagen synthesis (10, 12). In this study, the superior outcomes observed with the combined therapy may be related to the interaction of these biological mechanisms. Increased oxygen availability enhances the metabolic activity of fibroblasts and potentiates the regenerative effects of HA, enhancing the deposition of type I and III collagen and improving mucosal trophism. Furthermore, oxygen-rich microenvironments support neovascularization, counteracting the hypoxic milieu characteristic of GSM (11). The coordinated enhancement of angiogenesis and collagen biosynthesis likely explains why the combined approach outperforms treatment with hyaluronic acid alone. These remarks are consistent with previous reports showing that topical oxygen can potentiate the biological activity of HA and accelerate mucosal healing in atrophic gynecological tissues (10, 11). Although additional mechanistic studies are needed, particularly at the molecular level, the present findings support the hypothesis that LMWHA and oxygen therapy activate complementary pathways involved in tissue regeneration, offering a promising therapeutic strategy for women with GSM.

A study published in 2018 by Condemi et al. demonstrated how the treatment with LMWHA solution and HCO combined by a specific medical device is useful in the treatment of urinary symptoms related to GSM (11). In the diagnosis and management of mild and moderate stress or mixed urinary incontinence of GSM, the gynecological evaluation includes an accurate medical history and the use of validated questionnaires that investigate the presence, frequency, and impact of the symptoms on the quality of life; an accurate objective examination that searches for signs at the vulvar and vaginal level; and a request for tests and/or diagnostic procedures that confirm the diagnosis. Once the patients were selected, they were followed for 10 weeks of treatment, and at the end, they were re-evaluated with the same questionnaires administered at the beginning of the treatment. In this retrospective study, patients with severe stress urinary incontinence were not considered, since in this case, the therapy is strictly surgical (20). The use of the medical device Caress Flow© appears to constitute a valid treatment for GSM, especially useful for urinary symptoms of mild and moderate stress and mixed incontinence, being completely natural, well tolerated by patients with immediate therapeutic effects, with statistically significant improvement in symptoms before and after treatment, with recovery of one’s sexuality and urinary control, and without side effects. In this study, the first endpoint, according to the between-group analyses of the PGI-I questionnaire, was that post-menopausal women with vulvovaginal atrophy, suffering from symptoms of mild and moderate stress or mixed urinary incontinence, reported a subjective improvement of their symptoms at a significantly higher percentage when treated with LMWHA solution and HCO combined than with HA. There is no biological or pharmacological reason for believing that HA could exert any significant positive or negative effect on stress and mixed urinary incontinence symptoms; its use alone for this indication may be comparable to a placebo, unless it is associated with a combined treatment with HCO, in which an improvement in all questionnaire scores was seen in the post-treatment. Therefore, our data suggest that the Caress Flow© device might exert a beneficial effect on the symptoms of urinary urge incontinence in postmenopausal women with vulvovaginal atrophy. The medical device Caress Flow© had no contraindications or immediate side effects, had effective results perceivable from the first treatment, was a pleasant and non-invasive device, and had sessions that were very short (15 min) and did not require recovery time. The evaluation of secondary endpoints led to interesting observations. No difference in the AOB-S and ICIQ-SF total score was detected between the two groups at the beginning of the study, whereas after 10 weeks, it was significantly lower for the ICIQ-SF in the LMWHA solution and HCO combined group and HA group. Women using the combined LMWHA and HCO solution had a significantly better quality of life at the end of the study. However, this observation is hardly relevant, as the IQoL questionnaire score was already significantly higher at the beginning of the study in women who would use the LMWHA and HCO combined solution. In conclusion, our study showed a significantly superior effect of LMWHA solution and HCO combined with the medical device Caress Flow© over the use of only HA in improving the symptoms of mild and moderate stress or mixed urinary incontinence, the functional and inflammatory state of the vaginal mucosa, and, above all, the quality of life, which agrees with the previous literature. Furthermore, the combination of LMWHA solution and HCO represents one of the better alternatives for patients who do not want to take hormones, who have contraindications, or who have had negative experiences with hormone therapies.

This study presented several methodological limitations that must be acknowledged. First, the retrospective design inherently restricts the ability to control data collection and introduces the possibility of incomplete or non-uniform documentation. Second, the non-randomized nature of the study may contribute to selection bias, as the allocation of patients to treatment groups was not controlled or blinded. Third, the absence of randomization limits our capacity to fully account for potential confounders that may have influenced clinical outcomes. Although multivariable comparisons were performed, residual confounding cannot be excluded. Finally, because treatment assignment was based on clinical judgment rather than predefined criteria, differences in baseline characteristics across groups may have affected the observed results. These limitations should be considered when interpreting the findings, and future prospective, randomized controlled trials are needed to validate and strengthen our conclusions.

Taking into account that the therapy is innovative and requires outpatient access compared to a more comfortable and well-known home therapy, more patients must be enrolled to increase the sample size and increase the statistical power of our preliminary data or confirm it in a controlled design study, but these first results are very encouraging.

## 5 Conclusion

The current multicenter retrospective study supports the hypothesis that combined therapy with LMWHA solution and HCO, along with the Caress Flow© device, might improve the severity of mild or moderate stress and mixed urinary incontinence in this set of women. However, these results need to be confirmed in further studies with a controlled design and a larger population.

## Data Availability

The data analyzed in this study is subject to the following licenses/restrictions: the datasets used and/or analyzed during the current study available from the corresponding author on reasonable request. Requests to access these datasets should be directed to Basilio Pecorino, eliopek@gmail.com.
